# DNA origami-designed 3D phononic crystals

**DOI:** 10.1515/nanoph-2023-0024

**Published:** 2023-05-17

**Authors:** Sung Hun Park, Haedong Park, Jwa-Min Nam, Yonggang Ke, Tim Liedl, Ye Tian, Seungwoo Lee

**Affiliations:** KU-KIST Graduate School of Converging Science and Technology, Korea University, Seoul 02841, Republic of Korea; School of Physics and Astronomy, Cardiff University, Cardiff CF24 3AA, UK; Department of Chemistry, Seoul National University, Seoul 08826, Republic of Korea; Department of Chemistry, Emory University, Atlanta, GA 30322, USA; Wallace H. Coulter Department of Biomedical Engineering, Georgia Institute of Technology and Emory University, Atlanta, GA 30322, USA; Faculty of Physics and Center for NanoScience (CeNS), Ludwig-Maximilians-University, Geschwister-Scholl-Platz 1, 80539 Munich, Germany; College of Engineering and Applied Sciences, Nanjing University, Nanjing 210023, China; Department of Integrative Energy Engineering, Department of Biomicrosystem Technology, and KU Photonics Center, Korea University, Seoul 02841, Republic of Korea

**Keywords:** complete 3D phononic bandgap (PnBG), DNA origami, phononic crystals (PnCs)

## Abstract

Moulding the flow of phononic waves in three-dimensional (3D) space plays a critical role in controlling the sound and thermal properties of matter. To this end, 3D phononic crystals (PnCs) have been considered the gold standard because their complete phononic bandgap (PnBG) enables omnidirectional inhibition of phononic wave propagation. Nevertheless, achieving a complete PnBG in the high-frequency regime is still challenging, as attaining the correspondingly demanded mesoscale 3D crystals consisting of continuous frame networks with conventional fabrications is difficult. Here, we report that a DNA origami-designed-3D crystal can serve as a hypersonic 3D PnC exhibiting the widest complete PnBG. DNA origami crystallization can unprecedentedly provide 3D crystals such that continuous frame 3D crystals at the mesoscale are realizable. Furthermore, their lattice symmetry can be molecularly programmed to be at the highest level in a hierarchy of symmetry groups and numbers, which can facilitate the widening of the PnBG. More importantly, conformal silicification can render DNA origami-3D crystals rigid. Overall, we predict that the widest hypersonic PnBG can be achieved with DNA origami-designed 3D crystals with optimal lattice geometry and silica fraction; our work can provide a blueprint for the design and fabrication of mesoscale 3D PnCs with a champion PnBG.

## 1 Introduction

Phononic crystals (PnCs) enable exotic engineering of mechanical lattice vibration modes, promising compelling advantages in sound and thermal management such as sound shields [1], acoustic rectifiers [2, 3], acoustic superlenses [4, 5], acoustic waveguides [6], optomechanical cavities [7, 8], and insulating thermal dissipation materials [9–12]. Over the last two decades [13], materialization of 3D PnCs has undergone considerable progress from achieving a complete and broadband 3D phononic bandgap (PnBG) to pushing the frequency window of the PnBG into a higher regime [14]. In principle, efficient scattering of a mechanical wave and the subsequent collective interference are the critical ingredients for widening the width of the PnBG (*δ* = (*ω*_max_ − *ω*_min_)/[(*ω*_max_ + *ω*_min_)/2], where *ω*_max_ and *ω*_min_ correspond to the upper and lower frequencies of the PnBG, respectively) [15–18]. To this end, the local resonance of mechanical waves needs to be minimized as much as possible; consequently, a 3D crystal with a continuously connected frame rather than the discontinuous or bicontinuous counterparts has been preferred for PnBG materials [17, 18].

In the initial stage of their development, relatively low-frequency PnCs, i.e., working at Hz–kHz (corresponding to sound waves), have been mainly studied because their required unit cell scale can be readily addressed with a vast variety of fabrication methods common in daily life (e.g., hand-made assembly, 3D printing, and milling) [5, 19, 20]. However, to push the working frequency to GHz–THz (corresponding to the hypersonic and thermal regimes), 3D PnCs should have a mesoscale unit cell from a few hundred to tens of nanometres [14], which would be difficult to achieve with the aforementioned methods or common photolithography. Although electron beam lithography or focused ion beam lithography was used for the development of THz PnCs with a period of a few tens of nanometres, this monolithic lithography is not compatible with the engineering of a 3D continuous lattice [11, 12]. Meanwhile, interference lithography and colloidal assembly allowed fabrication of 3D PnCs with submicron unit cells [21–28]. However, these methods are generally limited to a discontinuous or bicontinuous 3D network [21–27]. Self-assembly of a block copolymer (BCP) provides a versatile platform for precise control of mesoscale (less than 50 nm) and sophisticated (e.g., double diamond, plumber’s nightmare, and Neovius) 3D crystal lattices [29–31]. However, their accessible crystals are also limited to a bicontinuous 3D network, which could, in turn, restrict the available upper limit of *δ* [32].

Only recently has 3D crystallization of DNA origami contributed to expanding the accessible lattice geometries of mesoscale and continuous 3D networks (e.g., the rod-connected 3D crystal) [33–35]. Unlike other molecular self-assembly methods (e.g., BCP or colloidal self-assembly), DNA origami can achieve a nearly arbitrarily shaped and sub-100 nm-scale building block (∼megadalton scale) [36, 37]. Note that this “nanoshaping” of DNA origami is available with a real molecular resolution (i.e., a precision of 0.33–0.34 nm in duplex length control and 2.0–2.5 nm in duplex width control) [38]. Furthermore, their hierarchical assembly into clusters and crystals (∼up to the gigadalton scale) has recently become accessible [33], [34], [35, 39]. Thus, crystallization of well-defined DNA origami enables unprecedented design and fabrication of a continuous 3D network at the mesoscale.

In this study, we theoretically show that DNA origami-designed 3D PnCs can exhibit a complete PnBG with a record-high *δ* in the hypersonic regime. First, we set the libraries of rod-connected continuous 3D networks composed of triangle and octahedral DNA origami. Herein, notably, the otherwise impossible engineering of mesoscale crystals is possible with 3D DNA origami. For example, isotropic and anisotropic DNA origami can be co-crystallized, which in turn enables precise control of the degree of lattice symmetry. Then, we numerically exploit the atlas of phononic band structures available with these 3D DNA origami crystals and systematically correlate the accessible *δ* with the order of the lattice symmetry. Generally, higher lattice symmetries lead to a wider *δ*. Finally, by taming the lattice periodicity and silicification of 3D DNA origami crystals, we computationally obtained a complete PnBG with a *δ* of 0.52, which was previously out of reach. Overall, the DNA origami approaches outlined in this work can provide integrative pipelines for the design and fabrication of mesoscale 3D PnCs with a champion *δ*.

## 2 Results and discussion

### 2.1 Design of DNA origami 3D PnCs

**Figure 1:**
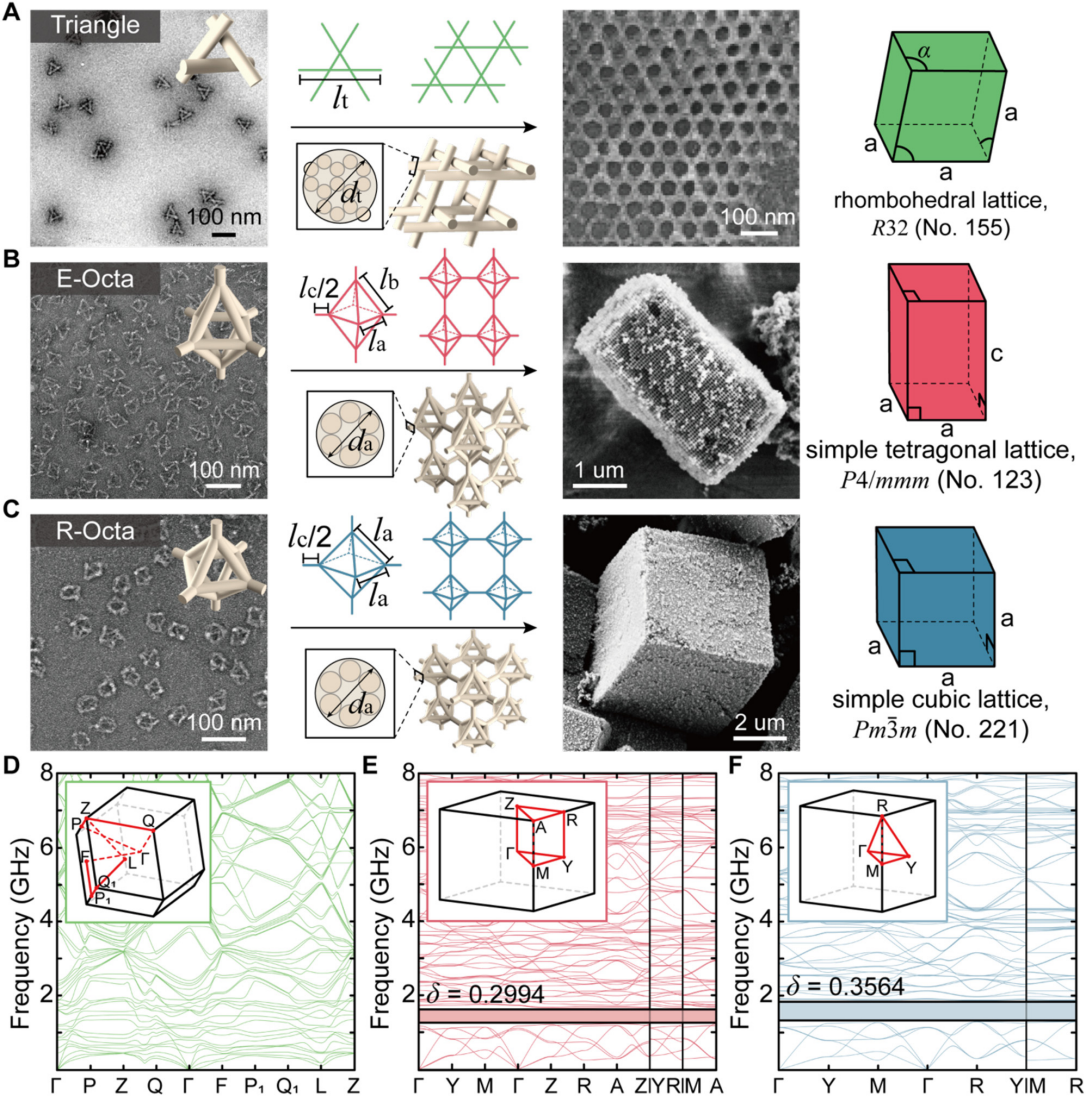
Representative examples of DNA origami 3D crystals that have been achieved and their phononic band structures. (A–C) Scheme and transmission electron microscopy (TEM) and scanning electron microscopy (SEM) images of (A) triangle, (B) E-Octa, and (C) R-Octa structures. Each has a particular Bravais lattice, such as a rhombohedral lattice (RL), a simple tetragonal lattice (STL), and a simple cubic lattice (SCL), respectively. Here, we use $a=\sqrt{2}l_{a}+l_{c}$ and $c=2\sqrt{l_{b}^{2}-l_{a}^{2}/2}+l_{c}$ in B and C, respectively. (D–F) Phononic band structures for A–C, respectively. Their BZ are also attached as insets. Note that the complete PnBG is advanced with increasing geometrical symmetry from the RL to the SCL. *δ* represents the relative bandwidth. All TEM/SEM images are adapted with permission from ref. [33] for panel A and ref. [34] for panel B–C.

To consider the experimental viability of 3D DNA origami, we start by defining the libraries of 3D DNA origami crystals. The experimentally fabricable 3D DNA origami crystals, shown in Figure 1A–C, are basic motifs for designing 3D PnCs throughout this study. Even though the advent of DNA origami can be traced back to 2006 [36], their 3D crystallization was achieved only 5 years ago. T. Liedl et al. [33] for the first time assembled triangle DNA origami into 3D crystals (i.e., a rhombohedral lattice (RL) of *R*32, no. 155; presented in Figure 1A). Each strut of the triangle consists of 14 DNA duplexes in a honeycomb lattice (14 helix bundles (HBs), shown in the cross-section image of Figure 1A). Later, Y. Tian et al. [34] successfully expanded the available library of DNA origami to be crystallized (Figure 1B–C). Particularly, they used both elongated (Figure 1B) and regular (Figure 1C) octahedral (E-Octa and R-Octa) DNA origamis to obtain a simple tetragonal lattice (STL of *P*4/*mmm*, no. 123; see macroscopic crystal habits in Figure S2) and a simple cubic lattice (SCL of *Pm* $\bar{3}$ *m*, no. 221; see macroscopic crystal habits in Figure S3), respectively. The octahedral frame is composed of 6 DNA duplexes (clustered into a honeycomb lattice (6 HBs), shown in the cross-section image of Figure 1B and C). Note that such recently materialized 3D DNA origami crystals all retain a rod-connected continuous 3D network. Other than these libraries, DNA origami has not been crystallized thus far. The structural data shown in Figure 1A–C were obtained for this work according to the protocols reported previously [33, 34].

We also should consider the DNA origami’s structural rigidities. In general, aqueous solution (buffer solution)-based assembly and 3D crystallization of DNA origami have been the prevalent protocol because the B-form DNA duplex, which is a basic motif of DNA origami, can maintain its structural integrity in a water medium. For these water-dispersed 3D crystals, however, the density of the DNA origami cannot be in stark contrast with that of the water medium, which in turn limits the accessible *δ*. This is because water molecules can readily penetrate the molecularly defined structural characteristics of the DNA origami crystals (i.e., major or minor grooves of the B-form DNA duplex) [40]. In contrast, only recently was an assembled DNA origami crystal experimentally proven to be able to maintain lattice fidelity even in ambient air [41–44].

Under this realistic condition, we first numerically verified the phononic band structures of DNA origami 3D crystals standing in the air. As with the previous reports on finite-element-method (FEM)-based mechanical analyses [45], each frame of the DNA origami was approximated by a single cylinder. The width of this cylinder was tuned according to the number of HBs. In particular, triangle (14 HBs) and octahedron (6 HBs) have different widths of the frame or strut, as shown in Figure 1A–C. Given the experimental results, the dimensions of triangle and octahedrons used in this study were set as follows: cross-sectional diameter of the triangle (*d*_t_) = 12.5 nm, length of each strut of the triangle (*l*_t_) = 67 nm, obtuse angle between adjacent edges (*α*) = 106°, cross-sectional diameter of octahedra (*d*_a_) = 6 nm, frame lengths of octahedra (*l*_a_ and *l*_b_) = 30 nm and 35 nm, and connector length between octahedra (*l*_c_) = 20 nm.

### 2.2 Importance of the lattice symmetry

The phononic band structures accessible with the air-standing 3D DNA origami crystals made of triangle (Figure 1A) and octahedrons (Figure 1B–C) are summarized in Figure 1D–F, respectively (more detail information for Brillouin zone (BZ) are included in Figure S4). The eigenfrequencies calculated along the paths in the BZ (reddish lines in the insets of Figure 1D–F) confirm that both octahedral STL and SCL can open a complete PnBG below 2 GHz (*δ* of 0.2994 for STL (Figure 1E) and *δ* of 0.3564 (Figure 1F) for SCL at 6–7 bands), while the tensegrity RL cannot retain a complete PnBG (Figure 1D). Note that octahedrons can also form an RL of *R* $\bar{3}$ *m*, no. 166 (see Section 2 in Supporting Information and Figure S1) and open a complete PnBG at 6–7 bands. Thus, we conclude that the octahedral crystallizations have advantages over the triangle crystallizations in terms of opening a complete PnBG. Note that the three rods in the triangle intersect each other at the lattice points, while all the rods in the octahedron are continuously connected without intersections. The intersected (triangle) and bulk continuous (octahedron) connections result in flexural and rigid vibrations of the phononic waves, respectively; the rigid vibration is principally better than the flexural counterpart in terms of opening a PnBG [18].

Additionally, it is noteworthy that the octahedral RL can exhibit a narrower *δ* (0.1688) than the STL and SCL at 6–7 bands (Figure S1), while the *δ* of the SCL is wider than that of the STL. Herein, we need to focus on points A and R of the STL (Figure S5A) and the SCL (Figure S5B) respectively, where *δ* reaches the minimum for both two PnBGs. These points are on the vertices of each BZ (see inset of Figure 1E–F). Point A in the STL has two- and four-fold symmetries without three-fold symmetry. The highest band at this point (represented as a green hollow dot and an arrow) is apart from the two degenerate bands (represented as black arrows) (Figure S5A). The three eigenstates at this point are not in three-fold symmetry; the eigenstate at the highest band possesses four-fold symmetry, while the other two eigenstates do not have four-fold symmetry, and only their relations are in four-fold symmetry. In contrast, point R in the SCL has two-, three-, and four-fold symmetries, and the three bands just below the PnBG are degenerated (Figure S5B). The three eigenstates at this point (represented as an orange hollow dot and arrows) are in three-fold symmetry, so their eigenfrequencies are the same. Thus, we can conclude that breaking the three-fold symmetry of R-Octa lifts a band towards the PnBG such that *δ* becomes narrowed. This result implies that a higher symmetry in 3D lattices gives rise to a wider *δ*.

**Figure 2:**
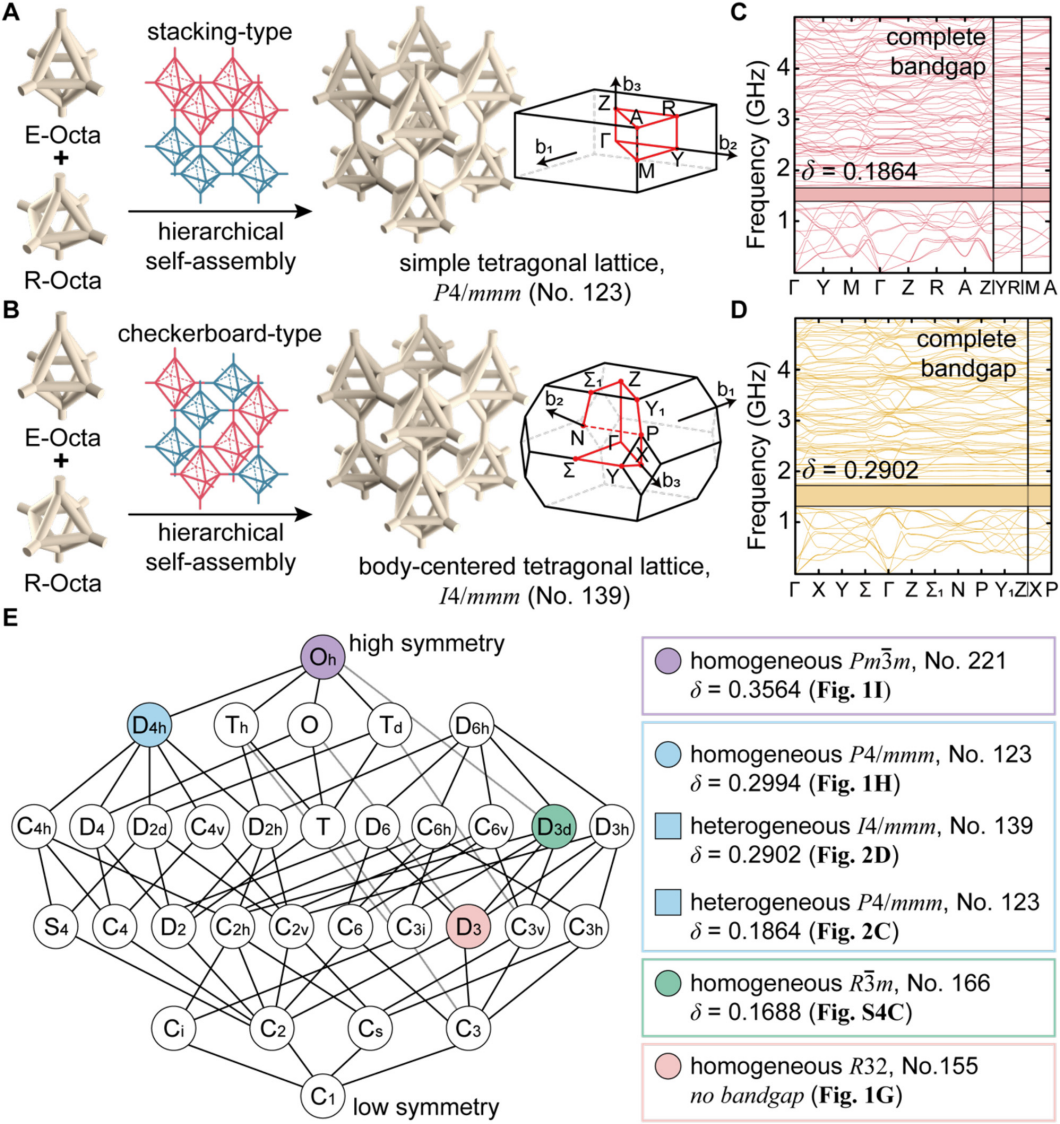
Cocrystallized DNA origami crystals consisting of E-Octa and R-Octa and their phononic band structures and hierarchical diagram representing the symmetry group of the DNA origami 3D crystals. (A–B) With differently programmed information at the end of protruding DNA, the final crystal motif can be represented as two different geometries, (A) stacking-type and (B) checkerboard-type. A and B correspond to a STL and a BCTL, respectively. (C–D) Phononic band structures for each crystal. (E) Hierarchical diagram of the symmetry group and number representing the relation between *δ* and symmetry.

The importance of the lattice symmetry for maximizing *δ* can be further emphasized by using multiscale assemblies of octahedral crystals. In contrast to BCP self-assembly, two different scale DNA origami can be cocrystallized, as shown in Figure 2A and B [35]. The protruding ends (single-stranded DNA sticky ends) of E-Octa and R-Octa can be programmed to be homogeneously crystallized in a 2D layer. Then, each 2D layer, where E-Octa and R-Octa are tetragonally crystallized, can be vertically stacked in an A–B–A–B type structure (i.e., a heterogeneous STL of *P*4/*mmm*, no. 123, as shown in Figure 2A). Each layer of the 3D crystal can also be designed to have a checkerboard-type array consisting of E-Octa and R-Octa and then stacked into a 3D body-centered tetragonal lattice (BCTL) of *I*4/*mmm*, no. 139 (Figure 2B). Herein, each layer can be vertically stacked with a 90° rotation angle. These cocrystallized TLs also intrinsically retain the lattice asymmetry, thus resulting in a narrower *δ* at 6–7 bands (*δ* of 0.1864–0.2902 in Figure 2C and D) compared to the *δ* of the symmetric cubic 3D lattice purely made of R-Octa (Figure 1F). Overall, the degree of lattice asymmetry exploited thus far can be hierarchically categorized according to the symmetry groups and numbers, as shown in Figure 2E; then, it can be directly correlated with the *δ* available at 6–7 bands. This result implies that higher lattice symmetry gives rise to a wider *δ*.

### 2.3 Silicification of DNA orgiami 3D PnCs

Such an upper limit of *δ* for DNA origami 3D crystals can be further increased by replicating DNA with harder materials. The Young’s modulus of the DNA duplex is low (300 MPa), leading to a relatively narrow *δ*. A versatile solution process can transform DNA duplexes into silica (i.e., a silicification process) [41–44], which provides otherwise impossible shaping or lattice engineering of silica. In particular, tetraethyl orthosilicate (TEOS) molecules can be conformally coated onto the minor and major grooves of DNA duplexes and chemically reduced with the assistance of ammonium. Thus, the homogeneous seeding of silica within DNA duplexes can indeed occur in a highly controlled manner; the subsequent growth of seeds can result in the formation of molecularly smooth silica frames on DNA origami templates. More importantly, the thickness of these molded silica frames can be precisely controlled simply by adjusting the reaction time [41–44]. As such, Young’s modulus of the frame considerably increases from 300 MPa (DNA helixes) to 70 GPa (silica), while the lattice geometry remains intact.

**Figure 3:**
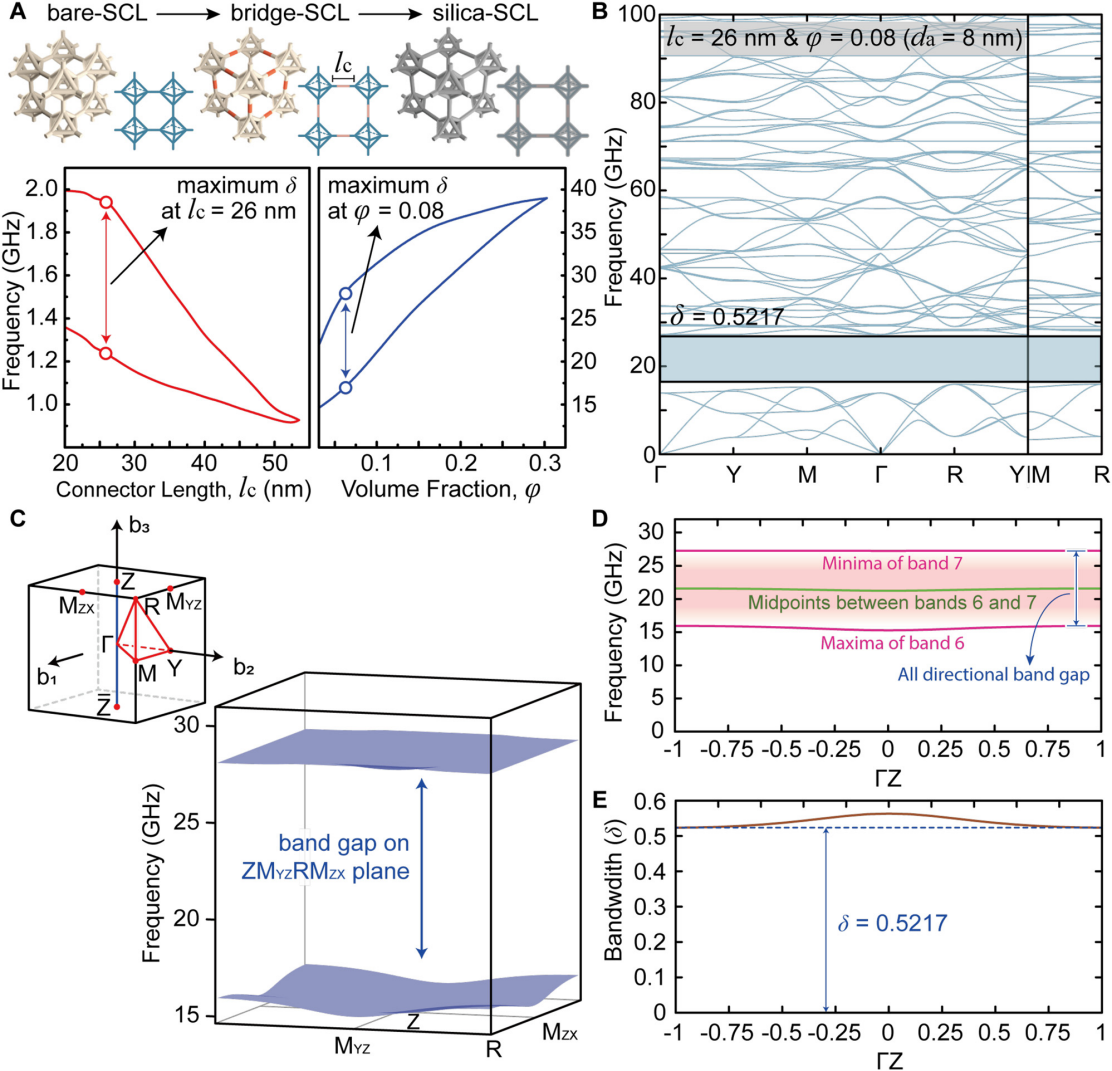
Bandgap tuning for the *δ* of SCL with respect to the connector lengths (*l*_*c*_) and the volume fraction of intercalated silica (*φ*). (A) (Top) schematic illustration of bare-, bridge-, and silica-SCL. (Bottom) the optimization for the *δ* of bridge-SCL with *l*_*c*_ and silica-SCL with *φ*. (B) 1D phononic band structure of the silica-SCL at optimal *l*_*c*_ (=26 nm) and *φ* (=0.08). (C) 2D phononic band structure (right-bottom) of the silica-SCL on the plane passing through *M*_*ZX*_-, *R*-, *M*_*YZ*_-, and *Z*-points in the BZ (left-top inset), related to the smallest bandwidth. (D) The minima of band 7 and the maxima of band 6 at the **b**_3_-normal slice planes at each point along the Γ*Z*-direction. Their averaged frequencies are also plotted as the green curve. (E) Bandwidth on the slice planes at each point along the Γ*Z*-direction.

We systematically verified the *δ* of DNA origami-designed silica crystals with respect to the connector lengths (*l*_c_) and the volume fraction of subsequently intercalated silica (*φ*) (Figure 3A). An increase in *φ* leads to an increase in *d*_a_ (the diameter of the cylinder). Note that these optimizations can be readily achieved with the currently viable DNA origami technology [41], [42], [43], [44, 46]. Herein, we focused on the bare-SCL (Figure 1C) because it can retain the widest *δ* among others (Figure 1D–F). At a *l*_c_ of 26 nm, *δ* of the bridge-SCL can reach the upper limits ranging from approximately 1–2 GHz at 6–7 bands (i.e., 0.4477). After silicification with a *φ* of 0.08 (*d*_a_ of 8 nm), *δ* of the silica-SCL can be further maximized (i.e., 0.5217) around 15–40 GHz at the same bands. The detailed phononic band structure at these optimal *l*_c_ and *φ* are depicted in Figure 3B. The phononic band structure of the STL with the optimized *φ* and *l*_c_ was also exploited, as represented in Figure S6; its accessible maximum of *δ* (0.4582) was lower than that for SCL (0.5217).

To confirm the complete PnBG of the silica-SCL at 6-7 bands, we further investigated the phononic band structure for all the *k*-points in the BZ (inset of Figure 3C). We prepared several **b**_3_-normal slice planes along the Γ*Z*-direction, and then calculated the eigenfrequencies on these planes. The minima of the band 7 and the maxima of the band 6 are almost consistent for all the k-points, as shown in Figure 3D, implying that the PnBG in Figure 3B is omnidirectional. The smallest PnBG comes from the plane passing through the *Z*-point (see Figure 3E). The band structure for this plane passing through the *M*_*ZX*_-, *R*-, *M*_*YZ*_-, and *Z*-points indicates that the maxima of the band 6 and minima of the band 7 arise at the *R*- and *Z*-points, respectively (see Figure 3C).

### 2.4 Analysis on the PnCs‘ vibration behaviours

**Figure 4:**
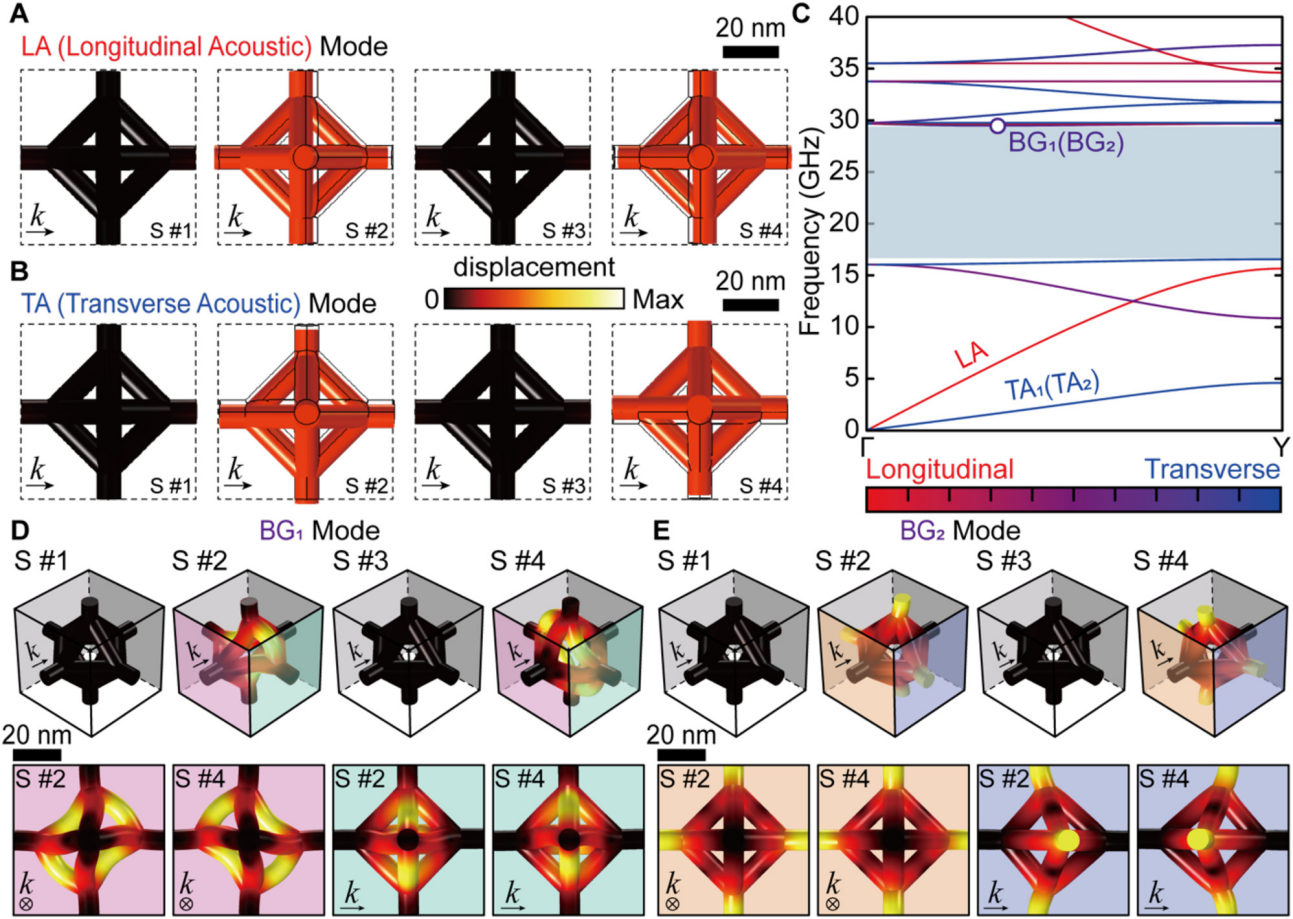
Modal analyses for the unit cell of the silica-SCL at the maximum *δ*. (A–B) Representation of modal analysis results for (A) LA and (B) TA modes. The dashed and solid line contours represent the undeformed configuration. In B, the other eigenstate is omitted due to the 4-fold symmetry. (C) Phononic band structure for the region of interest. Note that the Γ-*Y* direction corresponds to *k* = (0 1 0); Γ: *k* = (0 0 0) and *Y*: *k* = (0 1/2 0). The polarization fraction for an elastic wave is represented by the color, reflecting the values as encoded in the color map. The bluish color regions indicates the complete PnBG. (D–E) Representation of modal analysis results for (D) BG_1_ and (E) BG_2_ modes originating from wave interferences. The top and bottom rows present the perspective and orthogonal view, respectively. (A–B, D–E) Each mode is represented by the reconstructed deformation process (S #1 to S #4). All displacement amplitudes are normalized, with the same color bar.

From the above results, we extracted the eigenstates at selected points of the band structure to highlight their rigid vibrational modes. These modal analyses indicate how the DNA origami unit vibrates and which parts of the structure undergo relatively larger deformations. Figure 4 presents the corresponding modal analyses of the fundamental phononic modes in the unit cell of the optimally tuned the silica-SCL: we chose this the silica-SCL as a representative example because it can retain a maximum *δ* of 0.52. In particular, elastic plane wave propagations such as longitudinal acoustic (LA, Figure 4A) and transverse acoustic (TA, Figure 4B) modes are simultaneously excited from the Γ to *Y* point (Figure 4C). Herein, the phases of *ϕ*/2, *ϕ*, 3*ϕ*/2, and 2*ϕ* are referred to as S #1, 2, 3, and 4, respectively. With respect to the wavevector, harmonically induced lateral and vertical displacements of octahedrons with phases of *ϕ* and 2*ϕ* are visible for LA and TA modes, respectively (Figure 4A–B). Additionally, note that the slopes of the LA and TA modes in the band structures, branching from the Γ point, imply the group velocities for each polarization. The LA mode is found to be faster than the TA_1_ and TA_2_ modes. In the case of the TA mode, the two eigenstates (i.e., TA_1_ and TA_2_) are degenerate with four-fold symmetry around the propagation axis.

At the edges of the PnBG (see the upper bound of band structures in Figure 4C), such displacements of octahedral units are not observed, evidencing prohibition of elastic plane wave propagations. Instead, harmonic distortion of each octahedron is induced without displacement, resulting from the constructive interference of scattered elastic waves and the resultant formation of standing phononic waves. These standing phononic waves appear as two modes, denoted BG_1_ and BG_2_ (Figure 4D–E). The BG_1_ mode shows mirror-symmetric deformation of the octahedron along the out-of-plane direction with respect to the wavevector (distortions along the diagonal axis and off-diagonal axis with phases of *ϕ* and 2*ϕ*, respectively), while the BG_2_ mode results from two-fold symmetric deformation of the octahedron around the wavevector direction.

Notably, the key to achieving a wider *δ* is to induce efficient deformation of each octahedron unit in the BG modes. The silica-SCL composed of the solid octahedrons exhibits a narrower *δ* (0.2579) than the frame octahedral silica-SCL (Figure S7). This is because the solid octahedron is too rigid to be effectively deformed in the BG modes. The real-time behaviour of such modes in terms of the displacements, deformations, and energy fluxes is included in Supplementary Movie 1.

**Figure 5:**
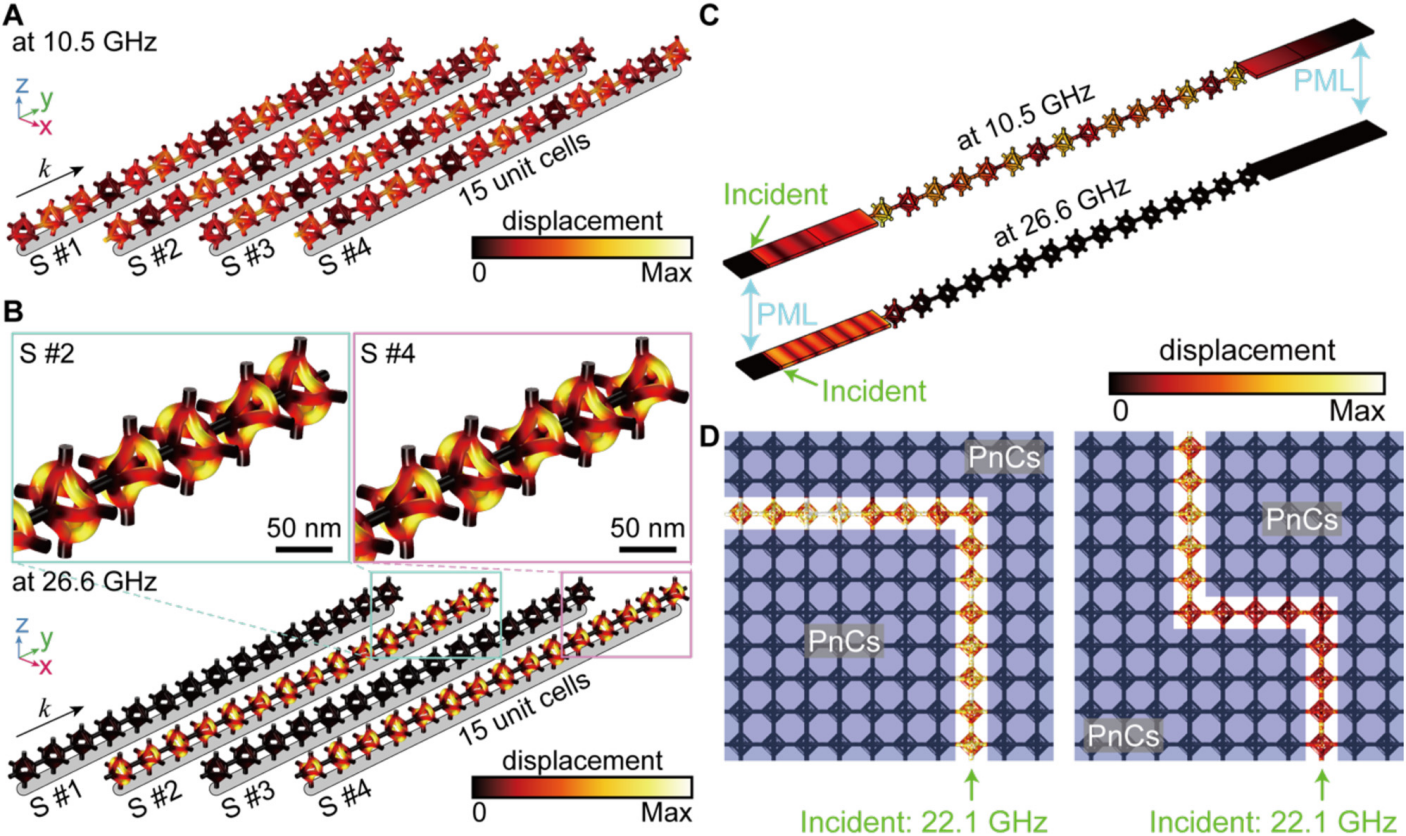
Modal analyses of uni- or multi-directional propagation in silica-SCL. (A–B) Deformation shapes in free standing 15-unit cells. (A) Outside the bandgap frequency, the incident waves can propagate without any disturb. (B) At the bandgap frequency, the incident waves are completely prohibited in the silica-SCL. The adjacent unit cells have out-of-phase deformations, resulting the standing wave in the BG_1_ mode. *k* represents the propagation direction of the elastic wave. Each arrays are represented by the reconstructed deformation process (S #1 to S #4) (C) The 1D wave propagations in unit cell arrays connected to the perfectly matched layer (PML)-terminated waveguide at outside (top) and within (bottom) bandgap frequencies. (D) The 2D waveguide combined with the bare-SCL and the silica-SCL. The incident wave propagates through the bare-SCL as a pathway and inhibited by surrounded the silica-SCL as waveguide blocks.

Figure 5 shows the corresponding modal analyses along the propagation direction for the free-standing 15-unit cells, which can further elucidate the modal characteristics of the elastic plane wave propagations. Outside the bandgap frequency (i.e., 10.5 GHz), elastic plane waves can readily propagate throughout the silica-SCL, as presented in Figure 5A. Regardless of the phase, distinct displacements of individual unit cells are observable. In contrast, propagation of elastic plane waves at the bandgap frequency (i.e., 26.6 GHz for the BG_1_) is completely prohibited in the entire network of the silica-SCL (Figure 5B). In particular, with phases of *ϕ* and 2*ϕ*, out-of-phase deformations between adjacent octahedrons are confirmed, evidencing the constructively interfering deformations of neighboring octahedrons and the resultant formation of a standing wave in the BG_1_ mode. The detailed vibration shapes for displacements and energy fluxes in entire unit cells are included in Supplementary Movie 2. When two ends of such 15-unit cells are connected to the perfectly matched layer (PML)-terminated input and output waveguides, the propagation and prohibition of elastic plane waves are further highlighted outside and inside the bandgap frequencies, respectively (Figure 5C).

Then, we constructed the multi-domain systems consisting of the bare-SCL and the silica-SCL for verifying the 2D waveguide property (Figure 5D). Herein, bare-SCL in Figure 1F and silica-SCL in Figure 3B can serve as a propagation path (non-bluish region) and PnBG-based mirrors (bluish region), respectively, such that the lines of bare-SCL, surrounded by silica-SCL, can define the phononic waveguides. As a proof-of-concept demonstration, we designed the single bending (left panel of Figure 5D) and zigzag bending (right panel of Figure 5D) geometries of two phononic waveguides; it was visible that the incident phononic waves at the bandgap frequency (i.e., 22.1 GHz) can be guided just along the line path of the bare-SCL. These results imply that such distinct materials (i.e., the bare-SCL and the silica-SCL) can be expressed as a hypersonic waveguide device.

**Figure 6:**
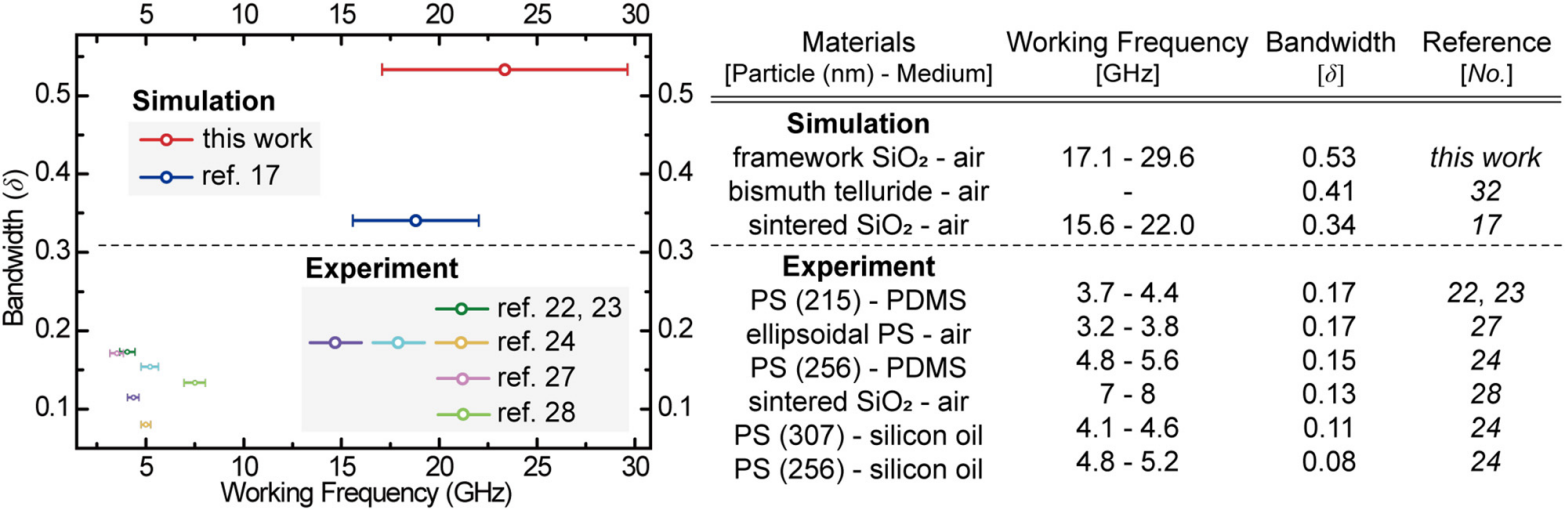
Comparison of bandwidths and working frequencies.

Last, we contrasted the *δ* metric of the DNA origami-designed PnCs with that of other counterparts reported thus far (Figure 6): the *δ* versus working frequency. We can conclude that the DNA origami-designed 3D PnCs can exhibit the widest *δ* at the highest frequencies ever achieved thus far both experimentally and theoretically. This outperforming *δ* of the DNA origami-designed PnCs can be attributed to their unique and exotic lattice characteristics, such as direct rod-connected continuous 3D networks at the mesoscale.

## 3 Conclusions

Taken together, we have suggested a DNA origami-designed 3D crystal as a versatile and programmable platform for achieving the widest *δ* of a complete PnBG in the hypersonic regime (tens of GHz). Nanoframes made of DNA origami can be assembled into mesoscale continuous 3D networks, which in turn make them advantageous over materials with other fabrication methods, especially for high-frequency 3D PnCs. The possible weakness of native DNA origami crystals, that is, their relatively low Young’s modulus, can be readily addressed by conformal silicification, which can also be viewed as a unique feature available with the DNA origami method. Even if currently available size of DNA origami crystal is relatively small (less than tens of micrometers), it is still effective for a chip-scale control over a high-frequency phononic wave [7, 11]. Overall, such a synergistic combination of DNA origami assembly and silica templating paves the way for unprecedented engineering of mesoscale 3D PnCs, consequently allowing us to reach the widest *δ* in the highest frequency regime, which was out of reach before this work.

## Materials and methods

The detailed simulations methods are in Section 1–3 in Supporting Information.

## Supplementary Material

Supplementary Material Details
