# Supplementary material for: DNA origami-designed 3D phononic crystals
Source: Nanophotonics. 2023 May 17;12(13):2611–21. doi: 10.1515/nanoph-2023-0024 (PMC11501301; doi:10.1515/nanoph-2023-0024)
Supplement: Supplementary file 1 — Supplementary Material Details [file j_nanoph-2023-0024_suppl_003.pdf]

Supporting Information for
DNA Origami-Designed 3D Phononic Crystals

Sung Hun Park, Haedong Park, Jwa-Min Nam, Yonggang Ke, Tim Liedl, Ye Tian, and Seungwoo Lee

Seungwoo Lee
Email: seungwoo@korea.ac.kr

Haedong Park
Email: ParkH5@cardiff.ac.uk

This PDF file includes:

1. Numerical calculation of phononic band structures
2. Deformation process of the regular octahedral DNA origami to fit in the rhombohedral lattice
3. Getting the full band gap information shown in **Fig. 3C-E**
4. SEM images of the 3D DNA origami crystals
5. Relevant data about phononic band structures

Other supporting materials for this manuscript include the following:

Movies S1 to S2

1. Numerical calculation of phononic band structures

For the simulation unit cell, the following eigenfrequency equation (S1) and Floquet boundary conditions (S2) were adjusted:

$$-\rho\omega^2\boldsymbol{u} - \nabla\cdot\boldsymbol{\sigma} = \boldsymbol{F}_v \quad \text{(S1)}$$

$$\boldsymbol{u}_2 = \boldsymbol{u}_1 e^{i\boldsymbol{k}_f\cdot(\mathbf{r}_2-\mathbf{r}_1)} \quad \text{(S2)}$$

where $\boldsymbol{u}$ is the displacement vector in the eigenstates, ρ is the density of the cylinders, ω is the eigenfrequency (angular frequency, $\omega = 2\pi f$), $\boldsymbol{\sigma}$ is the Cauchy stress, and $\boldsymbol{F}_v$ is the volume force. $\boldsymbol{k}_f$ and $\mathbf{r}$ represent the wavevector and position vector, respectively. Herein, subscripts 1 and 2 represent the indices of boundaries that define the periodicity of the 3D structure. In all simulations, the unit cell's size and shape were determined by geometrical parameters (e.g., l_a, l_b, l_c, l_t), as mentioned in the manuscript.

The elastic properties of the DNA duplex and SiO_2, used in the simulations, were as follows: Young's modulus of 300 MPa, Poisson's ratio of 0.48, and the density of 1.6450 g/cm^3 for the DNA duplex; Young's modulus of 70.55 GPa, Poisson's ratio of 0.17, and density of 2.41 g/cm^3 for SiO_2. All the simulations were carried out using commercial finite element method (FEM) software (COMSOL Multiphysics).

To identify the nature of the phonon wave, the displacement vector was written as $\boldsymbol{u} = \boldsymbol{u}_L + \boldsymbol{u}_T$ where $\boldsymbol{u}_L$ and $\boldsymbol{u}_T$ are its longitudinal and transverse components, respectively. The longitudinal component was expressed as $\boldsymbol{u}_L = (\boldsymbol{k}\cdot\boldsymbol{u}/\|\boldsymbol{k}\|^2)\,\boldsymbol{k}$, while the transverse component was given by $\boldsymbol{u}_L = \boldsymbol{u} - (\boldsymbol{k}\cdot\boldsymbol{u}/\|\boldsymbol{k}\|^2)\,\boldsymbol{k}$. The ratio of the longitudinal component to the transverse component was then written as $\langle\boldsymbol{u}_L|\boldsymbol{u}_L\rangle/(\langle\boldsymbol{u}_L|\boldsymbol{u}_L\rangle + \langle\boldsymbol{u}_T|\boldsymbol{u}_T\rangle)$, where $\langle\boldsymbol{a}|\boldsymbol{b}\rangle$ corresponds to $\int_{cell}(\boldsymbol{a}^*\cdot\boldsymbol{b})d^3\boldsymbol{r}$. This ratio was quantitated as reddish to bluish colours, as presented in **Fig. 4C** in the main text.

2. Deformation process of the regular octahedral DNA origami to fit in the rhombohedral lattice

We prepared the rhombohedral lattice (RL) of the regular octahedral DNA origami (R-Octa) by the following affine deformation process. We considered the lattice vectors of our rhombohedron to be $\mathbf{a}_1$, $\mathbf{a}_2$, and $\mathbf{a}_3$. To deform a cube whose lattice vectors are given by $\mathbf{A}_1$, $\mathbf{A}_2$, and $\mathbf{A}_3$, we prepared the following deformation gradient $\mathbf{F}$ that transforms the cube to a rhombohedron:

$$\mathbf{F} = \sum_{j=1}^{3}\mathbf{a}_j \otimes \widehat{\mathbf{A}}_j \quad \text{(S3)}$$

where $\widehat{\mathbf{A}}_j$ is the unit vector of $\mathbf{A}_j$. In realistic conditions, if an R-Octa in a cubic cell is mechanically stressed, then the deformation is not given by a simple affine transformation, and nonaffine behaviour would be observed due to the nonuniform distribution of material stiffness throughout the space. The same explanation can also be applied to the elongated octahedral DNA origami (E-Octa). However, we assumed that the difference between the affine and nonaffine deformations would be negligible. Thus, we used the affine deformation.

The preparation of the RL can be decomposed into the extension/compression along $\mathbf{A}_i$-directions and rotations. In other words, the deformation of a cube (and an R-Octa in this cube) to an RL can be described by a composition of only these extensions/compressions and rotations. This approach would be useful to prepare the RL of R-Octa if direct implementation of the above equation is impossible. To this end, we used RU decomposition as follows. First, the deformation gradient $\mathbf{F}$ is decomposed into a product of the principal stretch tensor $\mathbf{U}$ and the rotation tensor $\mathbf{R}$; $\mathbf{F} = \mathbf{RU}$. We should obtain each tensor. The right Cauchy-Green deformation tensor $\mathbf{C}$ is defined as $\mathbf{C} = \mathbf{F}^T\mathbf{F} = \mathbf{U}^2$. Thus, through its spectral decomposition $\mathbf{C} = \sum_{i=1}^{3}\lambda_i^2(\mathbf{N}_i\otimes\mathbf{N}_i)$, we obtain $\mathbf{U} = \sum_{i=1}^{3}\lambda_i(\mathbf{N}_i\otimes\mathbf{N}_i)$ and $\mathbf{R} = \mathbf{F}\mathbf{U}^{-1}$. Here, the eigenvalues λ_i and eigenvectors $\mathbf{N}_i$ ($\mathbf{N}_i\cdot\mathbf{N}_j = \delta_{ij}$) of $\mathbf{U}$ are called the principal stretches and principal directions, respectively.

Now, let us remember that we deal with the RL. Its lattice vector magnitudes are the same ($\mathbf{a}_i\cdot\mathbf{a}_i = a^2$), and all the angles between them are the same ($\mathbf{a}_i\cdot\mathbf{a}_j = a^2\cos\theta$). If we use $[\mathbf{A}_1, \mathbf{A}_2, \mathbf{A}_3] = I$, then, the right

Cauchy-Green tensor component C_{ij} of the above equation is expressed as $C_{ij} = a^2\{\delta_{ij} + (1-\delta_{ij})\cos\theta\}$. This tensor has multiple roots $\lambda_i^2 = 1-\cos\theta$ and a single root $\lambda_i^2 = 1+2\cos\theta$. Then, we used the single root λ_s and the corresponding eigenvector $\mathbf{N}_s$ for the following steps.

We rotated the cube such that $\mathbf{N}_s$ coincides with the $[0,0,1]$-direction; the cube was rotated by $\cos^{-1}(\mathbf{N}_s \cdot [0,0,1])$ around $\mathbf{N}_s \times [0,0,1]$. Next, the rotated cube was stretched by λ_i along the $\mathbf{A}_i$-direction. In fact, this process generated the RL whose lattice constant and angle are what we aimed for. However, to fit the result to the lattice vectors $\mathbf{a}_i$ that we initially set, we carried out further steps. We inversely rotated this RL, i.e., rotated it by $-\cos^{-1}(\mathbf{N}_s \cdot [0,0,1])$ around $\mathbf{N}_s \times [0,0,1]$. Finishing this step means that the initial cube was stretched by λ_i along $\mathbf{N}_i$. Then, we inputted the rotation $\mathbf{R}$ by rotating the octahedral lattice by $\cos^{-1}\{(\mathrm{tr}\mathbf{R}-1)/2\}$ around $[R_{32}-R_{23}, R_{13}-R_{31}, R_{21}-R_{12}]$ to obtain the final result of $\mathbf{F} = \mathbf{RU}$.

Unlike the results for the tensegrity, the R-Octa deformed to the rhombohedron exhibits a phononic bandgap (PnBG), as shown in **Fig. S4**. Its $\delta = (\omega_{max} - \omega_{min})/\{(\omega_{max} + \omega_{min})/2\}$ was 0.17, relatively narrow compared to the δ of R-Octa or E-Octa, and this tendency also coincided with the degree of symmetry shown in **Fig. 2** in the main text.

The above equation does not guarantee incompressibility. If we calculated the band structures from two RLs, one that satisfies and one that does not satisfy $\det[\mathbf{A}_1, \mathbf{A}_2, \mathbf{A}_3] = \det[\mathbf{a}_1, \mathbf{a}_2, \mathbf{a}_3]$, then the frequency scales in these two band structures are different. However, the bandwidth regarding the bandgap in each band structure, $\delta = (\omega_{max} - \omega_{min})/\{(\omega_{max} + \omega_{min})/2\}$, remains the same because the frequency scale is inversely proportional to the lattice size.

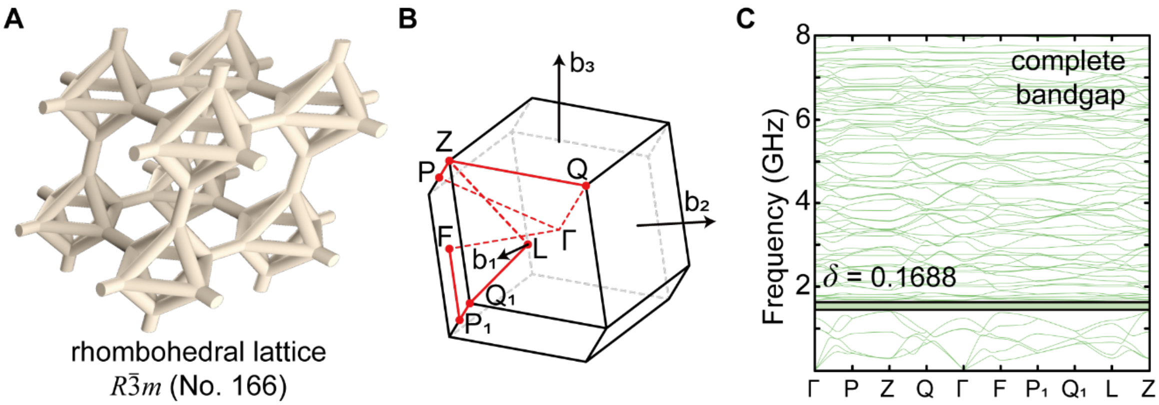

Fig. S1. (**A**) Schematic representation of deformed R-Octa in the RL and (**B**) the corresponding first Brillouin zone (BZ). (**C**) Corresponding phononic band structure. The volume of the primitive cell is adjusted to be the same as that for the SCL**.**

3. Getting the full band gap information shown in Fig. 3C-E in the main text

Along the ΓZ-path in the BZ (see the left-top inset of **Fig. 3C** in the main text), we set eleven points in equal spacing so that the path is divided into ten sections. At each point, we considered eleven square slice planes normal to $\mathbf{b}_3$-direction, As the R-Octa has a four-fold symmetry, all the results in $\mathbf{b}_1\mathbf{b}_2$ space are the same in the other three quadrants. So, we used the slice planes whose two edges are given by $\mathbf{b}_1/2$ and $\mathbf{b}_2/2$. We calculated equation S1 at 2500 points on each plane, then the maxima of the band 6 and minima of the band 7 of each plane was gathered, as shown in **Fig. 3D** in the main text. The bandwidth of each plane can be simply obtained, as shown in **Fig. 3E** in the main text. The R-Octa has a time-reversal symmetry. This means the eigenfrequencies in the BZ are inversion symmetric. Thus, we copied the results on the above planes passing the ΓZ-path to other planes passing the $\Gamma\bar{\mathrm{Z}}$-path.

4. SEM images of the 3D DNA origami crystals

Experimental samples on the 3D DNA origami crystals in simple tetragonal lattices (STL) and simple cubic lattice (SCL) are exemplified in Fig. 1. These show the experimental viability of the STL and SCL.

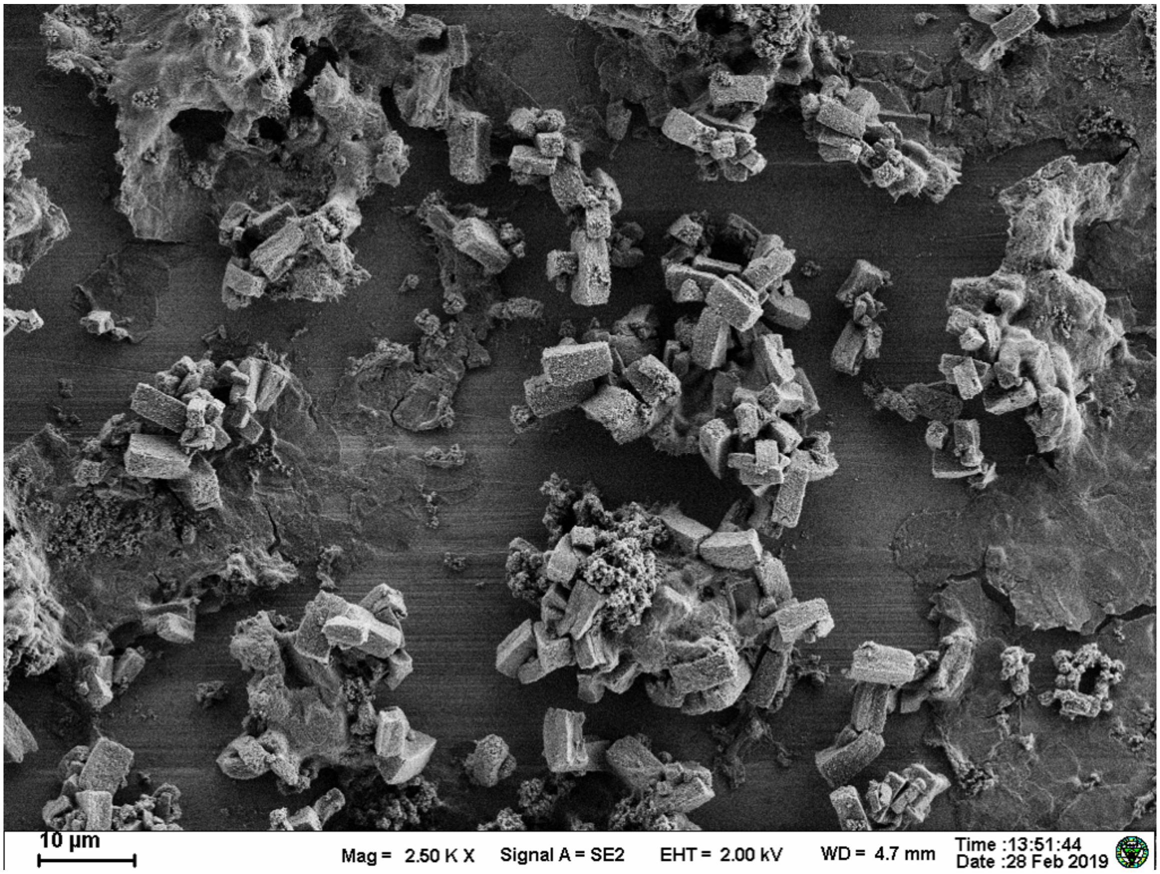

Fig. S2. SEM image of an STL, related to **Fig. 1B** and **E** in the main text.

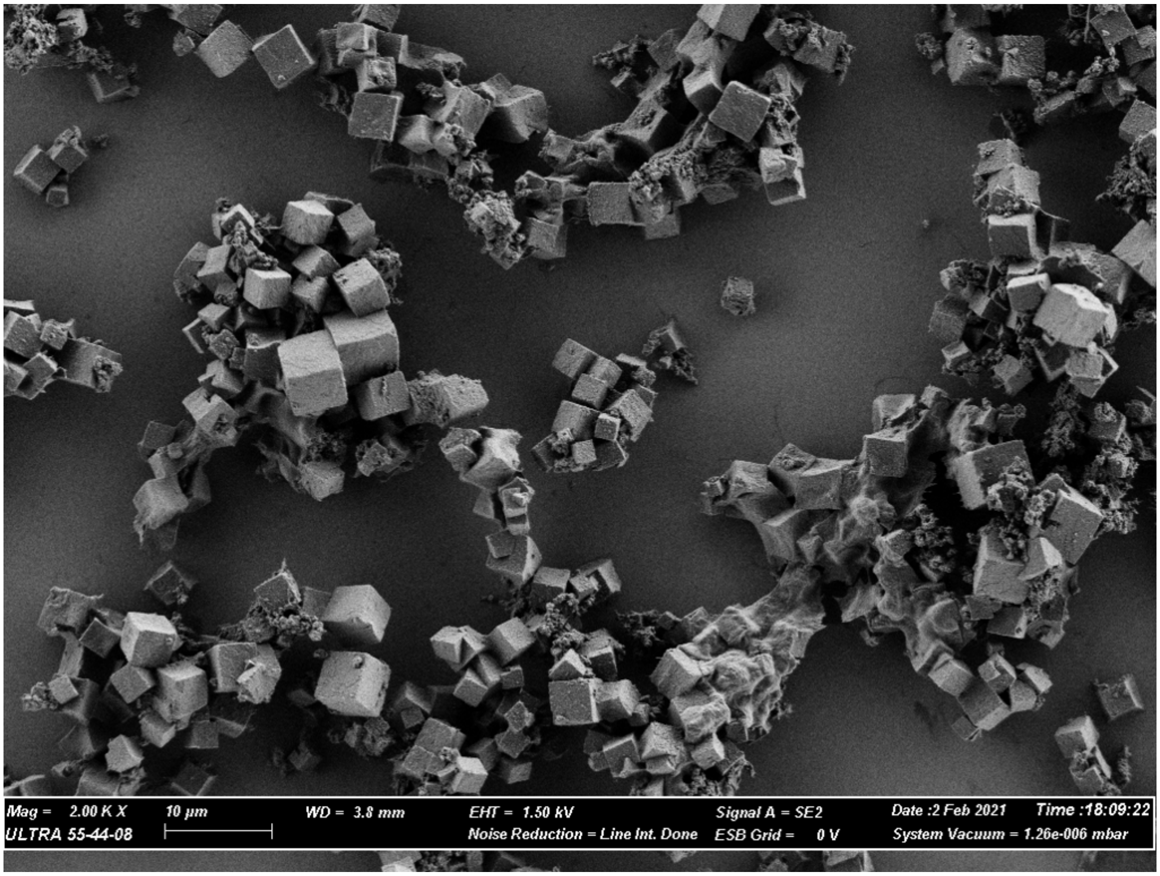

Fig. S3. SEM image of an SCL, related to **Fig. 1C** and **F** in the main text.

5. Relevant data about phononic band structures

A

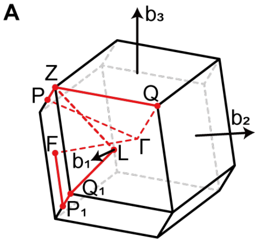

rhombohedral lattice
(Γ-P-Z-Q-Γ-F-P₁-Q₁-L-Z)

B

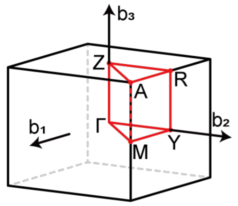

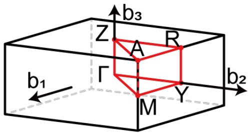

simple tetragonal lattice
(Γ-Y-M-Γ-Z-R-A-Z|Y-R|M-A)

C

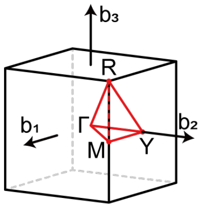

simple cubic lattice
(Γ-X-M-Γ-R-X|M-R)

D

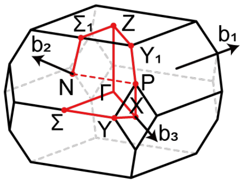

body-centered tetragonal lattice
(Γ-X-Y-Σ-Γ-Z-Σ₁-N-P-Y₁-Z|X-P)

E

	u(x**b**₁)	v(x**b**₂)	w(x**b**₃)
Γ	0	0	0
F	1/2	-1/2	0
L	1/2	0	0
P	1-υ	-υ	1-υ
P₁	υ	υ-1	υ-1
Q	η	η	η
Q₁	1-η	-η	-η
Z	1/2	-1/2	1/2

$\eta = 1/(2\tan^2(\alpha/2))$
$\upsilon = 3/4 - \eta/2$

F

	u(x**b**₁)	v(x**b**₂)	w(x**b**₃)
Γ	0	0	0
A	1/2	1/2	1/2
M	1/2	1/2	0
P	0	1/2	1/2
Y	0	1/2	0
Q	0	0	1/2

G

	u(x**b**₁)	v(x**b**₂)	w(x**b**₃)
Γ	0	0	0
M	1/2	1/2	0
R	1/2	1/2	1/2
Y	0	1/2	0

H

	u(x**b**₁)	v(x**b**₂)	w(x**b**₃)
Γ	0	0	0
N	0	1/2	0
P	1/4	1/4	1/4
Σ	-η	η	η
Σ₁	η	1-η	1-η
X	0	0	1/2
Y	-ζ	ζ	1/2
Y₁	1/2	1/2	-ζ
Z	1/2	1/2	-1/2

$\eta = (1+a'^2/c'^2)/4$
$\zeta = a'^2/(2c'^2)$

Fig. S4. BZ and high symmetry points of a (**A, E**) RL, (**B, F**) STL, (**C, G**) SCL, and (**D, H**) body-centered tetragonal lattice (BCTL). In (**E**), α = 106°. In (**H**), $a' = \sqrt{2}a$ and $c' = 2\lambda a$, where $\lambda = \left\{\left(2\sqrt{l_b^2 - l_a^2/2} + l_c\right) + \left(\sqrt{2}l_a + l_c\right)\right\}/\left\{2\left(\sqrt{2}l_a + l_c\right)\right\}$.

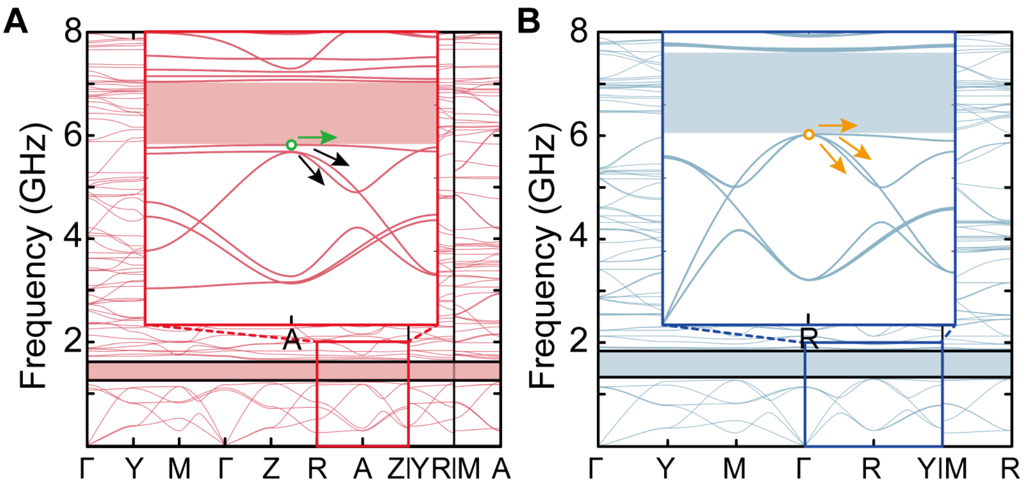

Fig. S5. Detailed analysis for symmetry points at (**A**) point A in the STL and (**B**) point R in the SCL. (**A**) The bands are split at point A which has no three-fold symmetry. These are represented as a green arrow and two black arrows. (**B**) The bands are degenerate at point R which has two-, three-, four-fold symmetries. These are represented as three orange arrows.

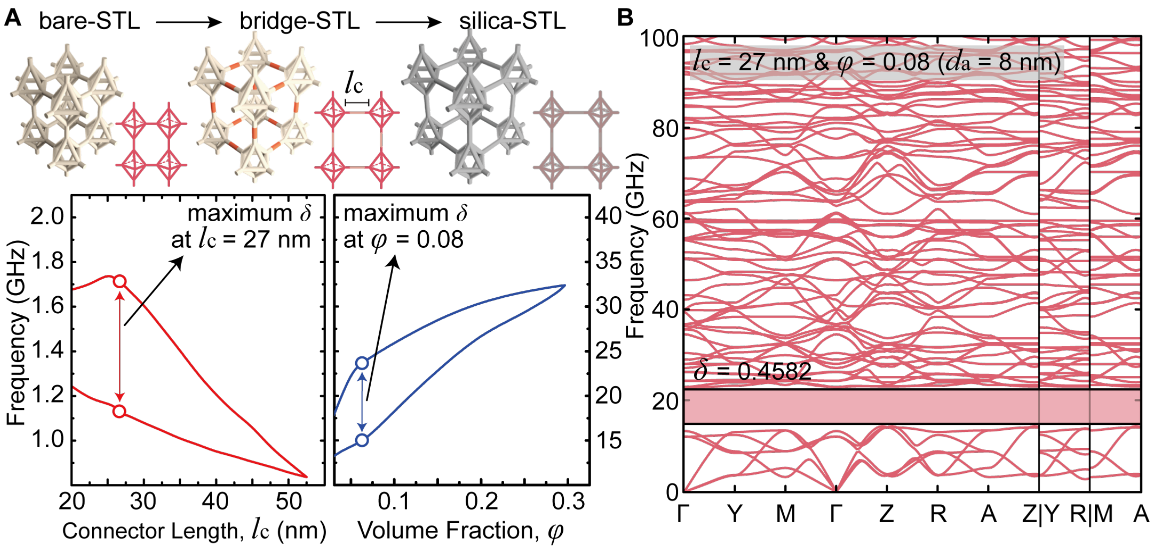

Fig. S6. Bandgap tuning for the δ of STL (E-Octa) with respect to the connector lengths (l_c) and the volume fraction of intercalated silica (φ). (**A**) (Top) schematic illustration of the bare-, bridge-, and silica-STL. (Bottom) the optimization for the δ of bridge-STL with l_c and the silica-STL with φ. (**B**) 1D phononic band structure of the silica-STL at optimal l_c and φ.

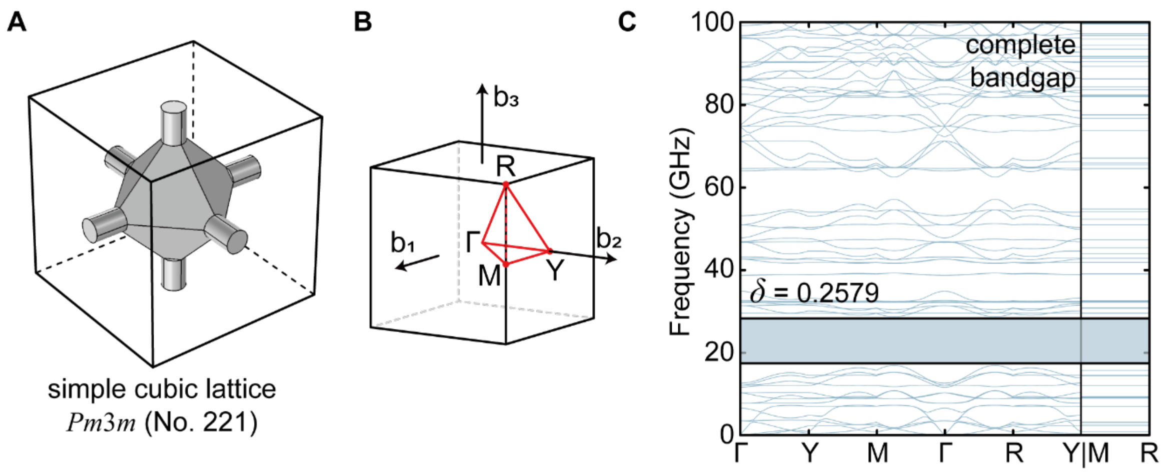

Fig. S7. (A) Schematic representation of solid silica R-Octa in the SCL and **(B)** the corresponding first BZ. **(C)** Corresponding phononic band structure.

Movie S1 (separate file). The vibration shapes for displacement and energy flux in TA, LA, BG_1, BG_2 modes.

Movie S2 (separate file). The vibration shapes for displacement and energy flux within bandgap frequencies (10.5 GHz, 39.8 GHz) and out of bandgap frequencies (26.6 GHz, 28.5 GHz)
